# The histone demethylase dLsd1 regulates organ size by silencing transposable elements

**DOI:** 10.1038/s42003-025-07724-6

**Published:** 2025-02-20

**Authors:** Ines Selmi, Manuela Texier, Marion Aguirrenbegoa, Clémentine Merce, Laurence Fraisse-lepourry, Bruno Mugat, Mourdas Mohamed, Séverine Chambeyron, David Cribbs, Luisa Di Stefano

**Affiliations:** 1 MCD, Centre de Biologie Intégrative (CBI), Université de Toulouse, CNRS, UPS, Toulouse, France; 2 Telethon Kids Institute, Nedlands, WA Australia; 3 Institute of Human Genetics, Université de Montpellier, CNRS, Montpellier, France

**Keywords:** Gene silencing, Limb development

## Abstract

The specific role of chromatin modifying factors in the timely execution of transcriptional changes in gene expression to regulate organ size remains largely unknown. Here, we report that in *Drosophila melanogaster* depletion of the histone demethylase dLsd1 results in the reduction of wing size. dLsd1 depletion affects cell proliferation and causes an increase in DNA damage and cell death. Mechanistically, we have identified Transposable Elements (TEs) as critical dLsd1 targets for organ size determination. We found that upon dLsd1 loss many TE families are upregulated, and new TE insertions appear. By blocking this new TE activity, we could rescue the wing size phenotype. Collectively, our results reveal that the histone demethylase dLsd1 and maintenance of TE homeostasis are required to ensure proper wing size.

## Abstract (web summary)

Genetic experiments coupled with genomic analysis reveal the role of the histone demethylase dLsd1 in regulating organ size via the repression of transposable elements’ transcription and mobilization in the model organism *Drosophila melanogaster*.

## Introduction

Understanding how organ size is controlled involves studying how cells grow, divide, die, and differentiate to form organs with a specific size and shape. This knowledge is fundamental to comprehending life. Perturbation of organ size can affect organ functions and is associated with many diseases, such as cancer, hypertrophic cardiomyopathy, renal hypoplasia and microcephaly^1,2^. The knowledge of how organs achieve and maintain their size is also required for regenerative medicine and tissue engineering. Wing development in *Drosophila melanogaster* is a classic model for understanding the genetic control of tissue size, shape, and patterning^3^. The wing’s final size depends on the cell capacity to grow, divide, and survive during development^3^. Most of the identified determinants of wing size belong to known and conserved signaling pathways, including the Hippo, JAK/STAT, TOR, and Insulin pathways^3^ that exert their effect through their interplay with key co-transcriptional activators (e.g.Yorkie), and transcription factors (e.g. scalloped and dmyc)^4–6^. However, little is known about the role of chromatin-modifying enzymes in the regulation of wing size. Here, we report that loss of function mutants of a critical chromatin modifier, the histone demethylase dLsd1 in *D. melanogaster* results in a reduction in wing size. LSD1 acts primarily as a co-repressor of transcription by demethylating mono and dimethyl histone H3 Lysine (K) 4 marks which are associated with enhancers and promoters^7,8^. In mice, LSD1 is essential for viability^9^ and is implicated in a wide variety of biological processes, including adipogenesis, spermatogenesis, and embryonic development^10–12^. In *D. melanogaster*, dLsd1 loss of function has been reported to result in oogenesis defect and sterility but a role in organ size determination had not been reported before^13,14^. In the ovary, dLsd1 regulates the expression of developmental genes, including genes involved in the cell cycle and plays a global role in TE silencing^15,16^. We, therefore, hypothesised that the reduction in wing size observed upon dLsd1 depletion in flies might be explained by its function as a co-transcriptional regulator of genes and TEs. TEs are mobile genetic elements that constitute around 20% of the *D.melanogaster* genome and up to 45% of the human genome^17^. Their ability to move within a genome threatens genomic stability and could therefore affect cell viability and organ size. Consistently, aberrant TE transposition has been linked to infertility, cancer, and neurodegenerative diseases^18,19^. However, the effects of TE deregulation on the different aspects of development are still mostly unknown.

In this study, we report that upon dLsd1 depletion, wing size is reduced due to a decreased wing cell number. This reduction in organ size is not limited to wings and concerned also head and leg size. We demonstrated that dLsd1 depletion in wing discs negatively affects cell proliferation and increases DNA damage and cell death. To elucidate the mechanisms by which dLsd1 influences wing size, we performed RNA-Seq experiments and, in line with our hypothesis, we found upregulation of many TE families in dLsd1 depleted wing discs. We showed, for the first time, that loss of dLsd1 results in new TE insertions in the genome. Moreover, blocking retro-transposition contributed to rescuing the wing size defects observed in *dLsd1* mutants. Taken together, these results indicate that excessive TE activation, resulting from dLsd1 loss, affects organ size.

## Results

### Depletion of dLsd1 leads to decreased wing size

**Fig. 1:**
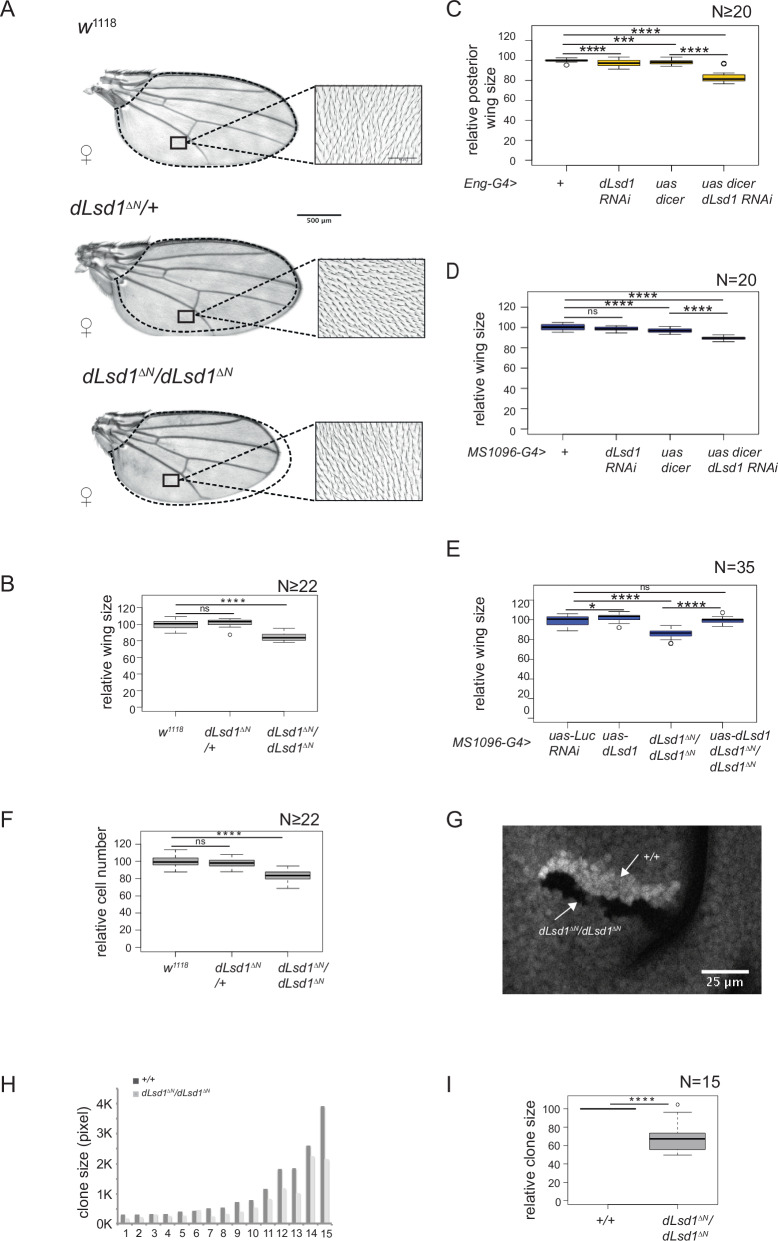
Wing size is reduced in adult flies harbouring a *dLsd1* loss of function mutation. **A** Images of wings and trichomes from *wild-type* (*w*^*1118*^), *heterozygous* (*dLsd1*^*ΔN*^*/+*) and *homozygous* mutant (*dLsd1*^*ΔN*^*/dLsd1*^*ΔN*^) females. The black dotted line marks the size of the *wild-type* wing for comparison. The black square represents the area of the wing used to quantify trichomes. Scale Bar: 50 µM (**B**) Quantification of the total wing areas in females of the indicated genotypes (*n* ≥ 22 wings per genotype). Data are relative to the *wild-type* value (*P* value < 0.001, ANOVA for all samples, and ns = not significant, ****P value < 0.001 unpaired *t*-test with Welch correction, for pairwise comparisons) (**C**, **D**) Quantification of posterior wing area (relative to controls) in females expressing RNA interference against dLsd1 in the posterior part of the wing using the Eng-GAL4 driver (**C**) or of the total wing area in females expressing RNA interference against dLsd1 in the wing pouch using the MS1096-GAL4 driver (**D**)**. E** Overexpression of dLsd1 rescues the wing size defect observed in *dLsd1*^*ΔN*^*/dLsd1*^*ΔN*^ adults. Quantification of the total wing area of *dLsd1*^*ΔN*^*/dLsd1*^*ΔN*^ and *wild-type* females expressing wild-type dLsd1 in the wing pouch (*MS1096-G4 driver*). Uas-Luc RNAi and *dLsd1*^*ΔN*^*/dLsd1*^*ΔN*^ flies were used as negative and positive controls respectively. *N* = 35 wings per genotype. (*P* value < 0.001, ANOVA for all samples, and, ****P value < 0.001, *P value < 0.05 unpaired *t*-test with Welch correction, for pairwise comparisons) (**F**) Cell number in wings from females of the indicated genotypes relative to *wild-type* control. N indicates the number of wings counted. (*P* value < 0.001, ANOVA, for all samples and ****P value < 0.001, ***P value < 0.01 unpaired *t*-test with Welch correction, for pairwise comparisons) (**G**) Representative image of a clone of *wild-type* cells (marked by two GFP copies) and its twin clone of *dLsd1*^*ΔN*^*/dLsd1*^*ΔN*^ mutant cells (marked by the absence of GFP) from a third instar larvae wing disc. Scale bar: 25 μM. **H**) Size distribution of individual *dLsd1*^*ΔN*^*/dLsd1*^*ΔN*^ mutant clones relative to their wild-type counterpart. *K* = 1000. **I** Quantification of *dLsd1*^*ΔN*^*/dLsd1*^*ΔN*^ mutant clone sizes relative to their wild-type counterpart (in percentage). *N* = 15 (****P value < 0.001, unpaired *t*-test with Welch correction).

We previously reported that a *dLsd1* null mutation, *dLsd1*^*∆N*^, results in wing vein patterning defects^13,14^. To further characterise dLsd1 role in wing development, we examined adult wings from flies homozygous for the null *dLsd1* mutant allele (*dLsd1*^*∆N*^*/dLsd1*^*∆N*^*)*. We observed a marked reduction in wing size in *dLsd1*^*∆N*^*/dLsd1*^*∆N*^ flies compared with *heterozygous* *dLsd1*^*∆N*^ (*dLsd1*^*∆N*^*/+)* and *wild-type* *w*^*1118*^ wings both, in females (15% decrease) (Fig. 1A, B) and in males (12% decrease) (Supplementary Fig. 1A, B), indicating that the size reduction is not gender-specific.

Given that dLsd1 depletion reduces male viability^13^, we decided to focus on females for the analysis of this phenotype.

We used complementary approaches to confirm that the observed reduction in wing size was due specifically to *dLsd1* depletion and not to other differences in genetic background between *dLsd1*^*∆N*^*/dLsd1*^*∆N*^ and *w*^*1118*^. First, we targeted RNAi expression to the posterior compartment of the wing discs using the *engrailed-GAL4* (*Eng-G4*) driver, and to the wing pouch giving rise to the wing proper, using the *MS1096-GAL4* driver. Targeted dLsd1 depletion in the posterior compartment of the wing disc and in the wing pouch led to a reduction in wing size of 17% and 10%, respectively (Fig. 1C, D and Supplementary Fig. 2A, B). Western blot analysis confirmed that dLsd1 protein levels were reduced upon RNAi expression (Supplementary Fig. 3A, B). Conversely, expressing wild-type dLsd1 in the *dLsd1*^*∆N*^ mutant wing pouch through the UAS-GAL4 system, reversed the wing size reduction associated with the *dLsd1* mutation (Fig. 1E). Finally, to alleviate concerns about possible effects of stock differences, we established isogenised *dLsd1*^*∆N*^/TM6B lines from individual males after outcrossing into a *w*^*1118*^ background. Homozygous mutants from these lines showed a wing reduction comparable to the original line used here (Supplementary Fig. 3C), indicating that the phenotype is not sensitive to its specific genetic background. Taken together, these results show that dLsd1 is required for normal wing size.

### dLsd1 cell autonomously regulates cell number in the wings

A difference in wing size could be due to changes in cell size, cell number, or both. To distinguish between these possibilities, we quantified cell densities in adult wings. As each epidermal cell in the wing secretes a single hair, we counted the number of hairs in a defined area of the wing (close up in Fig. 1A) and found that in wings of *dLsd1*^*∆N*^*/dLsd1*^*∆N*^ females, the cell number was reduced by 16%, while cell size was unchanged (Fig. 1F and Supplementary Fig. 1D). We obtained similar results in *dLsd1*^*∆N*^*/ dLsd1*^*∆N*^ males (Supplementary Fig. 1C, D) and upon RNAi-mediated knockdown of *dLsd1* (Supplementary Fig. 2C–F). These results show that the reduced wing size observed upon dLsd1 depletion is due to a decrease in the number of wing cells.

To determine whether this defect reflected a cell-autonomous requirement of dLsd1, we performed a clonal analysis to compare the growth of wild-type and *dLsd1*^*∆N*^*/dLsd1*^*∆N*^ cells in the same tissue using a classical FLP/FRT mitotic recombination approach^20^. Upon mitotic recombination between FRT sequences induced by conditional FLP expression driven by the heat-shock promoter in larvae, we obtained mosaic wing discs that harboured wild-type clones (+/+) marked positively by two copies of GFP, and their sister *dLsd1*^*∆N*^*/dLsd1*^*∆N*^ clones, marked by the absence of GFP (Fig. 1G). Comparison of the relative clone sizes of 15 twin spots, showed a marked decrease in the *dLsd1*^*∆N*^*/dLsd1*^*∆N*^ clone size (by 30%) relative to wild-type (Fig. 1H, I). We attributed this difference to dLsd1 function because in control experiments, mosaic wing discs harbouring wild-type twin clones marked by two copies of GFP or RFP, did not show any difference in the relative clone size (Supplementary Fig. 4A–C). These results indicate that dLsd1 acts cell autonomously to regulate cell number. To determine whether this dLsd1 function was specific to wings discs, we analysed the size of +/+ clones and their sister *dLsd1*^*∆N*^/*dLsd1*^*∆N*^ clones in eye, antenna and leg discs. In most cases, *dLsd1*^*∆N*^/*dLsd1*^*∆N*^ clones were significantly smaller than their twin +/+ clones (Supplementary Fig. 4D). Additionally, the head size and tibia length were both significantly reduced in *dLsd1*^*∆N*^/*dLsd1*^*∆N*^ flies compared to *wild-type* flies (Supplementary Fig. 4E, F). These results show that dLsd1 is required to achieve normal organ size in different somatic tissues. Thus, the effect on wing cells appears to reflect a general *dLsd1* function.

### dLsd1 loss perturbs cell cycle progression

**Fig. 2:**
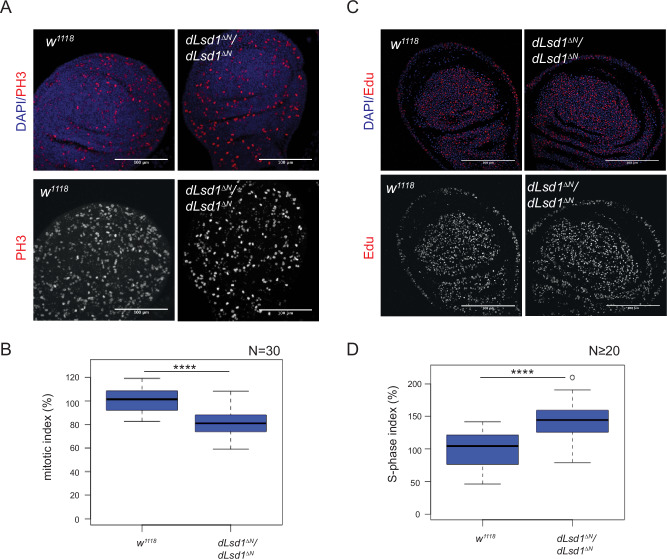
A *dLsd1* loss of function mutation negatively affects wing cell proliferation. **A** Images of *wild-type* and *dLsd1*^*ΔN*^*/dLsd1*^*ΔN*^ wing discs of feeding third instar females. Total maximum intensity-projections are shown. Discs were stained with DAPI to visualise DNA and with PH3 to mark cells in mitosis (Scale bar = 100 µM). **B** Quantification of the mitotic index of *wild-type* and *dLsd1*^*ΔN*^*/dLsd1*^*ΔN*^ wing pouches of female feeding L3. The mitotic index is the percentage of PH3 positive cells relative to *wild-type* control. Experiments were performed in triplicate. N indicates the number of wing discs counted. ****, *P* value < 0.001 (Student’s *t*-test). **C** Representative images of *wild-type* and *dLsd1*^*ΔN*^*/dLsd1*^*ΔN*^ wing discs of feeding third-instar females. Total maximum intensity projections are shown. DNA was visualised with DAPI and EdU incorporation was used to mark the nucleus of cells in S-phase (Scale bar = 100 µM). **D** Quantification of the S-phase indexes in *wild-type* and *dLsd1*^*ΔN*^*/dLsd1*^*ΔN*^ in the wing pouches of female feeding L3 wing discs. The S-phase index is expressed as the percentage of Edu-positive cells relative to *wild-type* control. Experiments were performed in quadruplicate. N indicates the number of wing discs counted. ****P value < 0.001 (Student’s *t*-test).

The decrease in cell numbers upon dLsd1 depletion might reflect cell cycle defects. Cell proliferation in the wing discs is mostly observed between the mid L3 stage and post-pupation, when it grows from about 1000 cells to roughly 50,000 cells per disc^21^. To assess whether the smaller wing size observed in *dLsd1*^*∆N*^*/dLsd1*^*∆N*^ mutants was due to a perturbation of the cell cycle, we first assessed the presence of phosphorylated histone 3 serine 10 (PH3), a mitotic marker, in wing discs at the feeding (mid-L3) and wandering stages (late L3). We detected an 18% and 14% reduction in the mitotic index (number of PH3-positive cells/wing pouch area) in mid-L3 and late L3 mutant discs, respectively, compared with *wild-type* discs (Fig. 2A, B and Supplementary Fig. 5A).

To further characterise the cell distribution in the cell cycle, we labelled cells with 05-ethynyl-2**′**-deoxyuridine (EdU), an alkyne-containing thymidine analogue that is incorporated into DNA during DNA replication. The number of EdU-positive cells was 42% higher in *dLsd1*^*∆N*^*/dLsd1*^*∆N*^ than in wild type discs (Fig. 2C, D), as indicated by the S/G2 phase index (number of EdU-positive cells/wing pouch area).

We then used RNAi driven by the *Eng-G4* driver to deplete dLsd1 in the posterior compartment of the wing disc and observed a small but significant increase in cells blocked in G2/M (Supplementary Fig. 6A–C).

Taken together, the decrease in the number of mitotic cells and the concomitant increase in S/G2-phase cells upon dLsd1 depletion in wing cells suggests that *dLsd1* loss of function affects cell proliferation rate by lengthening the S/G2 phase or blocking cells in the S/G2-phase and thus restricting their entry into mitosis.

### Altered cell cycle is associated with increased DNA damage and activation of the apoptotic machinery

**Fig. 3:**
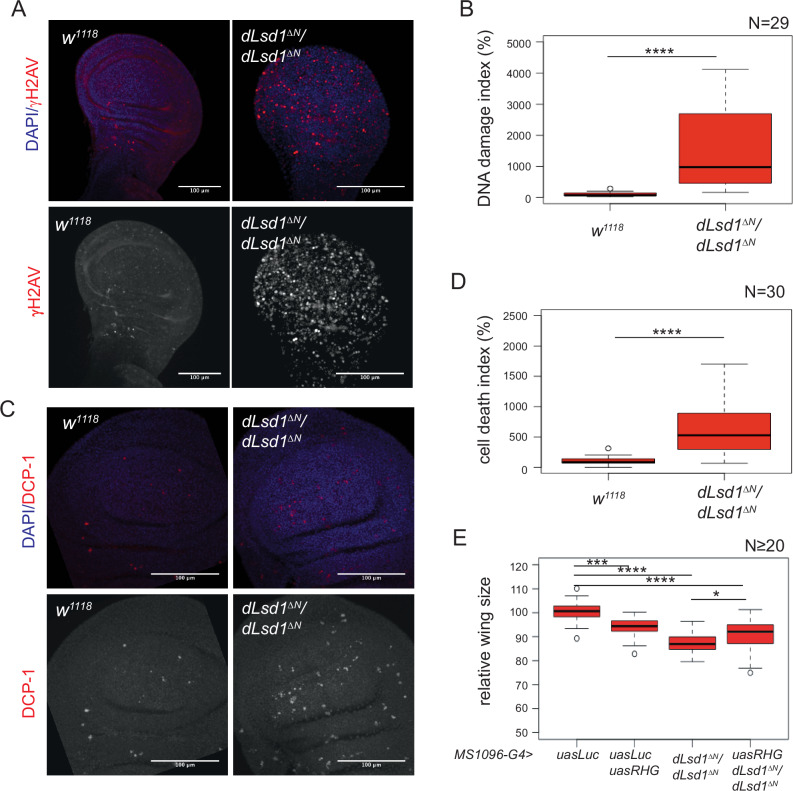
A *dLsd1* loss of function mutation induces DNA DSBs and activates the apoptotic machinery. **A** Representative images of *wild-type* and *dLsd1*^*ΔN*^*/dLsd1*^*ΔN*^ wing discs from feeding third-instar females. Total maximum intensity projections are shown. Discs were stained with DAPI to mark nuclei and with ɣH2Av to mark DNA DSBs (Scale bar = 100 µm). **B** Quantification of the DNA damage index in *wild-type* and *dLsd1*^*ΔN*^*/dLsd1*^*ΔN*^ wing pouches from feeding L3 females. The DNA damage index is expressed as a percentage relative to *wild-type* control. Experiments were performed in triplicate. N indicates the number of wing discs counted. ****, *P* < 0.001 (Student’s *t*-test). **C** Representative images of *wild-type* and *dLsd1*^*ΔN*^*/dLsd1*^*ΔN*^ wing discs from feeding third-instar females. Total maximum intensity projections are shown. Discs were stained with DAPI to visualise DNA and with DCP-1 to mark apoptotic cells (Scale bar = 100 µm). **D** Quantification of the cell death index in *wild-type* and *dLsd1*^*ΔN*^*/dLsd1*^*ΔN*^ in the wing pouches of feeding L3 females. The cell death index is expressed as a percentage relative to *wild-type* control. Experiments were performed in triplicate. N indicates the number of wing discs counted. ****P value < 0.001 (Student’s *t*-test)**. E** Quantification of the wing size in females of the indicated genotypes. N indicates the number of female wings counted. ****P value < 0.001, ***P value < 0.01, *P value < 0.05 (Student’s *t*-test).

The delay in cell cycle progression could reflect the activation of an S-phase checkpoint that senses DNA damage^22^. Therefore, we probed for DNA double-strand breaks (DSBs) using the marker ɣH2Av^23^ in mid to late L3 wing discs from *dLsd1*^*∆N*^/*dLsd1*^*∆N*^ and *wild-type* female. The percentage of ɣH2Av-positive cells was 15-fold higher in *dLsd1*^*∆N*^/*dLsd1*^*∆N*^ than *wild-type* wing discs (Fig. 3A, B and Supplementary Fig. 5B), indicating that DNA damage was strongly increased in *dLsd1*^*∆N*^*/dLsd1*^*∆N*^ wing discs.

DNA DSBs activate checkpoint mechanisms that can lead to cell cycle arrest or to apoptosis, in case of severe damage. The genetic programme leading to apoptosis involves the activation of initiator and then executioner caspases (cysteine proteases) by sequential cleavages. In *Drosophila melanogaster*, the Death Caspase 1 (DCP-1) is one of the main executioner caspases. Immunostaining of L3 wing discs for the cleaved activated form of Dcp1 to detect ongoing apoptotic cell death was 6-fold higher in *dLsd1*^*∆N*^/*dLsd1*^*∆N*^ compared to *wild-type* discs at the same developmental stage (Fig. 3C, D and Supplementary Fig. 5C). A significant increase in apoptosis was also observed when dLsd1 was depleted by RNAi in the posterior compartment and is not observed in control flies (Supplementary Fig. 7A–C).

The proteins Reaper (Rpr), head involution defective (Hid) and Grim, collectively termed RHG proteins, are critical inducers of apoptosis in *D. melanogaster*. Their removal is sufficient to strongly inhibit caspase-dependent apoptosis^24^. Therefore, we asked whether blocking apoptosis by inhibiting RHG protein expression could rescue wing size in *dLsd1*^*∆N*^/*dLsd1*^*∆*^ mutants. To this end, we targeted the *UAS-RHG* microRNA (miRNA), which generates miRNAs that simultaneously inhibit *reaper*, *hid*, and *grim* expression^25^ to the wing pouch using the *MS1094-GAL4* driver. The wing size reduction observed in *dLsd1*^*∆N*^/*dLsd1*^*∆N*^ mutants was alleviated by RGH inhibition, demonstrating an active role of apoptosis in wing size reduction (Fig. 3E).

Altogether, our results show that DNA damage and apoptosis are increased in *dLsd1*^*∆N*^/*dLsd1*^*∆N*^ wing discs. We infer that the dLsd1 loss makes cells more susceptible to DNA damage.

### RNA-Seq analysis reveals aberrant expression of metabolic genes and TEs in *dLsd1* mutant wing cells

**Fig. 4:**
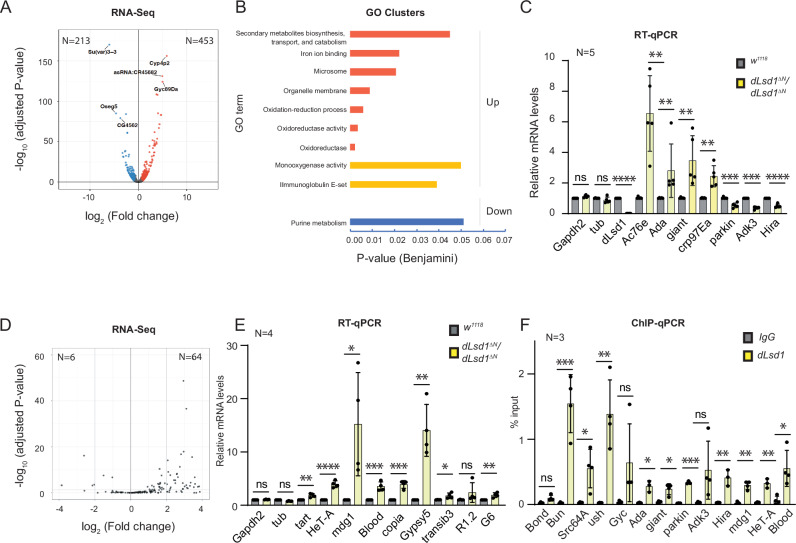
A *dLsd1* loss of function mutation affects the expression of metabolic genes and TEs. **A** Volcano plot showing the RNA-seq data of *wild-type* and *dLsd1*^*ΔN*^*/dLsd1*^*ΔN*^ wing discs from wandering third instar females. Blue and red dots mark genes downregulated and upregulated respectively, upon dLsd1 depletion (log2(FoldChange) > 0.6 and adjusted *p*value < 0.1). **B** Gene ontology (GO) analysis (DAVID 6.8) of genes upregulated or downregulated in *dLsd1*^*ΔN*^*/dLsd1*^*ΔN*^ wing imaginal discs compared to wild-type. The red, yellow and blue clusters represent genes with GO terms associated with oxidation-reduction processes, with immune response and with purine metabolism, respectively. Genes in the red and yellow clusters were upregulated, genes in the blue cluster were downregulated. **C** RT-qPCR analysis of the expression of the indicated genes using independent RNA samples from wild-type and *dLsd1*^*ΔN*^*/dLsd1*^*ΔN*^ wing discs from L3 females. α-*Tubulin* was used as control. The expression level was normalised relative to *wild-type* control and *Rp49* was used as a reference. Error bars indicate the standard deviation. N indicates the number of biological replicates. A student *t*-test was performed. **D** Volcano plot showing the RNA-seq data of *wild-type* and *dLsd1*^*ΔN*^*/dLsd1*^*ΔN*^ wing discs from wandering third instar females. Blue and red dots are used to mark the downregulated and upregulated TEs respectively (−1 > log2(FoldChange) > 1 and adjusted *p* value < 0.05). **E** RT-qPCR analysis of the expression of the indicated TEs using independent RNA samples from *wild-type* and *dLsd1*^*ΔN*^*/dLsd1*^*ΔN*^ female L3 wing discs. α-*Tubulin* and *Gapdh2* were used as controls. The expression level was normalised relative to *wild-type* control and *Rp49* was used as a reference. **F** ChIP-qPCR analysis of dLsd1 binding to a subset of deregulated genes and TEs in *wild-type* wing discs. IgG was used as a control for antibody specificity. *Bond* was used as a negative control and *Bun, ush* and *Src64A* were used as positive controls. Error bars indicate the standard deviation. N indicates the number of biological replicates. *P value < 0.05, **P value < 0.02, ***P value < 0.01, ****P value < 0.001 (Student’s *t*-test).

It has been shown that dLsd1 modulates gene expression in different organisms including *D. melanogaster*. To test whether dLsd1-dependent changes in gene expression might explain the wing phenotype, we sequenced RNA samples from wing discs of wandering third instar *wild-type* and *dLsd1*^*∆N*^/*dLsd1*^*∆N*^ female larvae and compared their transcriptomes. By applying a cut-off threshold of 0.6 for the log_2_ Fold Change and of 0.1 for the adjusted *p*value, we identified 666 genes that were differentially expressed in the *dLsd1*^*∆N*^/*dLsd1*^*∆N*^ samples (453 upregulated and 213 downregulated genes), (Supplementary Data 1 and volcano plot in Fig. 4A). Gene Ontology (GO) analysis showed that the genes upregulated in *dLsd1*^*∆N*^/*dLsd1*^*∆N*^ were enriched in GO terms associated with oxidation-reduction processes and immune response, while downregulated genes were enriched in GO terms associated with purine metabolism (Fig. 4B). Among the top 30 upregulated genes, we found genes coding for cuticular proteins, long-non coding RNAs and genes normally expressed in neurons. To validate the RNA-Seq results, we selected eight genes based on their fold change and implication in wing biology and performed RT-qPCR analysis using fresh RNA samples. In agreement with the RNA-Seq results, the expression of Ac76E, Ada, giant, Crp97Ea, Gyc-89Da was significantly increased, whereas the mRNA expression of parkin, Adk3 and Hira was significantly decreased in *dLsd1*^*∆N*^/*dLsd1*^*∆N*^ compared with *wild-type* samples (Fig. 4C and Supplementary Fig. 8A).

We previously showed that depletion of dLsd1 in ovaries results in TE de-repression^15^. To verify whether dLsd1-dependent TE silencing extends to somatic wing cells, we aligned our RNA-sequencing reads to a database of transposons sequences and performed a differential analysis. We found that 64 TE families were upregulated and 6 were downregulated in *dLsd1*^*∆N*^/*dLsd1*^*∆N*^ compared with *wild-type* wing disc samples (Fig. 4D and Supplementary Data 2) (log_2_ Fold Change 1, adjusted *p* value 0.05). We confirmed by RT-qPCR the de-repression of selected TEs in *dLsd1*^*∆N*^/*dLsd1*^*∆N*^ mutant wing discs compared to wild-type samples: LINE-retrotransposons HeT-A (3.9-fold) and G6-DM (1.9-fold), LTR retrotransposons, Gypsy5 (14-fold), Blood (3.5-fold) and mdg1 (15.2-fold) (Fig. 4E). The increase in TE expression in *dLsd1*^*∆N*^/*dLsd1*^*∆N*^ wing cells is likely not due to the difference in TE content between *w*^*1118*^ and the *dLsd1*^*∆N*^ mutant flies, because RT-qPCR, showed that the expression level of the subset of TEs examined was similar in *dLsd1*^*∆N*^/+ (heterozygous) and *wild type* wing discs (Supplementary Fig. 8B). Therefore, we conclude that TE de-silencing is a consequence of dLsd1 loss.

Then, we performed Chromatin Immunoprecipitations (ChIP) experiments followed by qPCR to determine whether dLsd1 directly binds to a subset of deregulated genes and TEs. We detected dLsd1 binding to the TSS region of the genes *Ada*, *giant*, *parkin* and *Hira* and to the TEs *mdg1*, *HeT-A* and *Blood* (Fig. 4F). We used *Bun*, *Src64B* and *ush* as positive controls, because they were previously shown to be bound by dLsd1 and *Bond* as negative control^15^.

Taken together, these data show that dLsd1 regulates gene and TE expression in wing discs. As TE activation has been linked to increased genome instability^26^, the upregulation of some TE families in *dLsd1*^*∆N*^/*dLsd1*^*∆N*^ wing discs might explain some of the phenotypes observed in the absence of dLsd1. Therefore, we focused on TEs.

### *dLsd1* loss in wing discs results in TE mobilisation

**Fig. 5:**
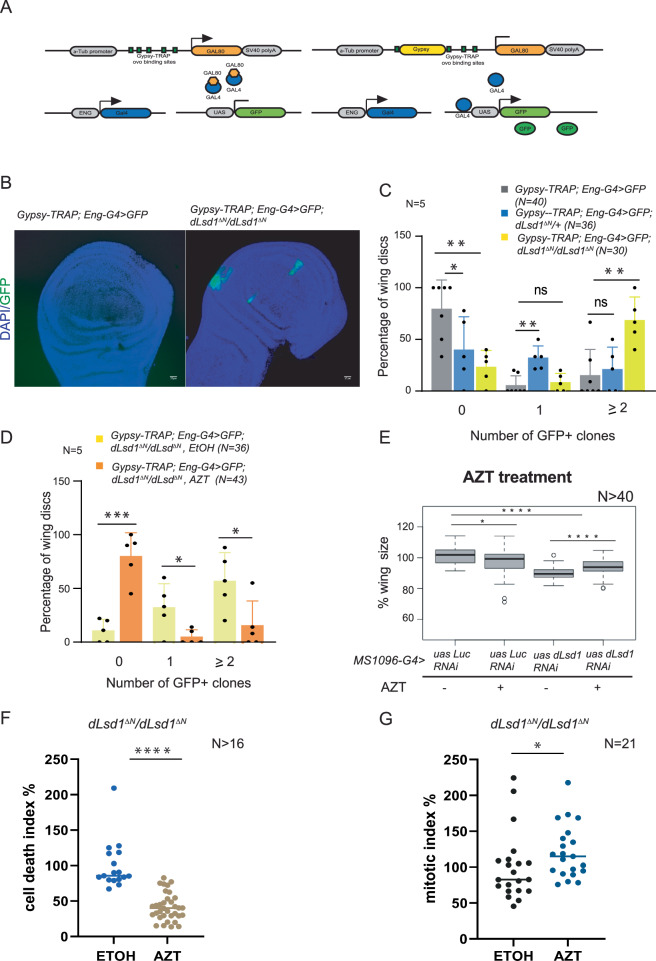
A *dLsd1* loss of function mutation results in activation of transposable elements. **A** Schematic representation of the Gypsy-TRAP mobilisation assay modified from Li et al.^19^. The insertion of Gypsy/mdg4 in the ovo binding sites prevents GAL80 expression thus allowing EngG4 driven GFP expression. **B** Representative images of wing discs without (*Gypsy-TRAP; Eng uasGFP*), and with (*Gypsy-TRAP; Eng uasGFP; dLsd1*^*ΔN*^*/dLsd1*^*ΔN*^) GFP positive clones indicative of Gypsy/mdg4 mobilisation events. Scale bar = 25 µm (**C**, **D**) Histograms showing the percentage of wing discs from flies of the indicated genotypes with 0, 1 or 2 or more transposition events. The experiment was repeated 5 times. N indicates the number of wing discs used for this analysis. The *P* value was calculated with the Student’s *t*-test. **E** Blocking retrotransposition partly rescues the wing size defect due to dLsd1 loss of function. Quantification of total wing areas in females of the indicated genotypes exposed to 2.5 mg/ml of AZT or Ethanol (control). The experiment was repeated five times. N indicates the number of wing discs used for this analysis. The *P* value was calculated with the Student’s *t*-test. **F** Quantification of the cell death index in *dLsd1*^*ΔN*^*/dLsd1*^*ΔN*^ treated or not with AZT. N indicates the number of wing discs counted. ****P value < 0.0001 (Mann–Whitney test). **G** Quantification of the mitotic index in *dLsd1*^*ΔN*^*/dLsd1*^*ΔN*^ treated or not with AZT. N indicates the number of wing discs counted. *P value = 0.02 (Mann–Whitney *U*-test).

The TE transcriptional derepression observed in *dLsd1*^*∆N*^/*dLsd1*^*∆N*^ wing cells could lead to TE activation and a higher frequency of transposition. We tested this hypothesis using a gypsy-TRAP reporter system that takes advantage of the preferential integration of the retrotransposon gypsy (now renamed mdg4) into the regulatory region of the *ovo* gene to detect de novo mobilisation events (Fig. 5A)^19^. We monitored these events in the wing discs by looking at *Eng-GAL4*-driven GFP expression in the posterior compartment (Fig. 5B). De novo insertion of Gypsy/mdg4 in the *ovo* binding sites (placed between the ubiquitous tubulin promoter and the coding sequence of the GAL4 inhibitor, GAL80) prevents the expression of GAL80, thus allowing-GAL4 driven GFP expression in the same cells. Cell proliferation following the integration resulted in clones of GFP-positive cells. We counted the number of GFP-positive clones in the posterior compartment to assess the occurrence of transposition events in *wild-type* wing discs compared to heterozygous and homozygous *dLsd1*^*ΔN*^ mutant wing discs. As shown in Fig. 5B, C, the percentage of wing discs with multiple GFP-expressing clones (indicating transposition events) increased from 9.5% in wild type to 32% in *dLsd1*^*ΔN*^/+ and 86% in *dLsd1*^*∆N*^/*dLsd1*^*∆N*^ mutants.

To confirm that the appearance of GFP-positive clones corresponded to integration events, we used zidovudine (AZT) to block reverse transcription, a requisite step of the Gypsy/mdg4 life cycle^27^. As shown in Fig. 5D, exposing the flies to 2.5 mg/ml of AZT blocked the appearance of GFP-positive clones, reflecting Gypsy/mdg4 integration, in *dLsd1*^*∆N*^/*dLsd1*^*∆N*^ wing discs.

These results indicate that dLsd1 loss leads to Gypsy/mdg4 transposition in wing discs. To determine whether this concerned also other TE families, we performed long-read DNA sequencing of wild-type and homozygous/heterozygous *dLsd1*^*ΔN*^ wing discs using the Oxford Nanopore Sequencing technology. We took advantage of the high quality of the *dLsd1*^*ΔN*^/+ fly library, to perform a telomere-to-telomere assembly of the two non-balanced chromosomes of this laboratory stock (chromosomes X and II). Using the TrEMOLO pipeline for TE annotation to map the long reads to the dLsd1 assembly^28^, we identified 285 and 117 TE insertional variants, in mutant and wild-type wing discs libraries respectively. We considered as newly inserted only the TEs that segregated at low frequencies (<8%) in the libraries as determined by a TrEMOLO module ^28^ and only families with at least two new insertions. This left 132 new insertions in *dLsd1*^*∆N*^/*dLsd1*^*∆N*^ wing discs, belonging to 28 TE families (Supplementary Data 3) and 19 new insertions belonging to 5 TE families in wild-type wing discs. This confirmed that transposition frequency was increased in *dLsd1* mutants compared with controls.

We then used zidovudine (AZT) to determine whether the mobilisation of retrotransposons contributed to the decrease in wing size observed upon dLsd1 depletion. Individuals expressing *MS1096-G4>uas-dicer; uas-dLsd1 RNAi or uas-LucRNAi* were fed with 2.5 mg/ml of AZT over the entire developmental period and the wing size was compared to that of flies of the same genotype in the absence of AZT treatment. As shown in Fig. 5E, AZT treatment led to a significant rescue of the wing size of *MS1096-GAL4 > uas-dicer; uas-dLsd1* RNAi flies, showing that the size reduction following dLsd1 depletion is at least partly explained by increased TE transposition. Additionally, the percentage of apoptotic cells in the wing pouch of *dLsd1*^*∆N*^/*dLsd1*^*∆N*^ L3 individuals is reduced upon treatment with AZT and the number of cells in mitosis increased (Fig. 5F, G), indicating that blocking retrotransposition is rescuing the cell cycle defect and the increased cell death in *dLsd1*^*∆N*^/*dLsd1*^*∆N*^ wing discs.

The piRNA pathway has been shown to play a major role in heterochromatin assembly, particularly on TE sequences in non-gonadal somatic cells in the early embryo, and failure of heterochromatin formation in the early embryo impacts adult phenotypes^29^. To determine if this role could impact the size of the wing discs, we measured the wing area following Piwi or Aubergine depletion by RNAi with or without dLsd1 co-depletion (Supplementary Fig. 9A). Depleting Piwi or Aubergine decreased the posterior wing area by 24% and 21%, respectively. Co-depleting dLsd1 decreased the posterior wing area by 26%, and 22%, respectively (not significant compared with Piwi and Aubergine depletion alone) (Supplementary Fig. 9A). Moreover, Piwi or Aubergine depletion decreased the posterior cell number by 9% and 18% and the posterior cell size by 16% and 4%, respectively, whereas dLSD1 co-depletion did not have any additional effect (Supplementary Fig. 9B, C). These results show that depletion of Piwi and Aubergine strongly affects wing size highlighting a new role for piRNAs pathway components in the early embryos impacting the somatic tissues of the adults.

Altogether these results indicate that regulation of TE homoeostasis is critical to achieve normal wing size.

## Discussion

In this study, we discovered that in *D. melanogaster* dLsd1 is implicated in the regulation of organ size through its role as a regulator of retrotransposon activity. Specifically, dLsd1 depletion affected cell cycle progression, caused increased DNA damage and cell death in wing discs and led  to global changes in gene expression and TE derepression. The increase in TE expression, due to dLsd1 depletion, was accompanied by TE mobilisation that affected wing size.

We found that dLsd1 depletion resulted in a significant reduction of wing size due to decreased cell number. Our RNAi experiments and mitotic clone experiments revealed two important facts. First, the reduction of wing size was cell-autonomous and not due to a systemic effect linked to dLsd1 depletion elsewhere in the body. This implies that normal dLsd1 levels are required for tissue  homeostasis. Second, this is a general property of dLsd1, not restricted to wing epithelial cells, because we observed the same effect in clones from other imaginal discs. Similarly, smaller adult wings were accompanied by narrower heads and shorter leg segments. This observation led to the question of whether dLsd1 depletion affects organ size by perturbing the cell cycle. Indeed, inhibition of LSD1 slows the proliferation of a subset of cancer cell lines^30,31^. Using specific markers, we demonstrated that dLsd1 depletion in Drosophila epithelial wing cells affected the cell cycle by increasing the number of cells in the S/G2-phase and decreasing the number of cells in mitosis. The increased S/G2 cell population observed in *dLsd1*^*ΔN*^ mutants could be due to the activation of an intra-S phase checkpoint that triggers replicative stress. Alternatively, it could reflect the activation of a G2 checkpoint. Replicative stress has been defined as a transient slowing of replication forks to ensure removal of DNA damage^32^ and it has been observed in different mammalian cell types and in Drosophila germline cells^33^ but not in wing cells. Therefore, an alternative explanation could be that, in *dLsd1* mutant cells, cell cycle progression is delayed through the activation of a G2 checkpoint, as previously described in wing cells upon ionising radiation-induced DNA damage^33^. Consistently, in *dLsd1*^*ΔN*^ mutants, the cell cycle perturbation was accompanied by increased DNA damage, as indicated by the higher number of γH2Av foci compared with wild type wings. The aberrant caspase activity observed upon *dLsd1* loss and the partial restoration of normal wing size in *dLsd1*^*ΔN*^ mutants by blocking critical components of the apoptotic pathway (hid, reaper and grim) show that apoptotic induction contributes to the decrease in wing size, most likely when DNA damage cannot be properly repaired. Taken together, these findings indicate that dLsd1 depletion makes wing cells more sensitive to DNA damage.

The RNA-Seq experiments provided insights into the mechanisms by which dLsd1 deregulation could induce genome instability and DNA damage in imaginal discs. Indeed, multiple TE families were upregulated in *dLsd1*^*ΔN*^ mutants. Additionally, by using the gypsy-TRAP reporter and long-read sequencing, we discovered that upon dLsd1 loss, TEs become more mobile. Two key findings are that both apoptosis and wing size defect caused by dLsd1 depletion were rescued by blocking retrotransposition. This indicates that TE mobility contributes to the reduction in wing size observed in *dLsd1* mutants. In agreement, depletion of two key TE regulators, Piwi and Aubergine also resulted in reduced wing size. This is likely due to their role in embryos as Gu and Elgin has shown that failure of Piwi-mediated heterochromatin assembly at TEs in embryos impacts heterochromatin homoeostasis at TEs in adult non-gonadal somatic cells^29^. Similarly, a very recent paper, showed that increased TE expression and mobility induced by knockdown of condensin subunits (key regulators of chromosome organisation) in *D. melanogaster* neural stem and progenitor cells lead to smaller adult head size and brain volume^34^. Altogether, these results provide compelling evidence that aberrant TE activation affects organ size. This is consistent with the fact that TEs are a major source of genetic instability. Indeed, to transpose TEs need to break the DNA to insert themselves in a new location in the genome. Often, these insertions are unsuccessful and constitute a source of DNA double-strand breaks^35^. Consequently, the increase in DNA double-strand breaks and cell death that we observe in dLsd1- wing discs could be due, at least in part, to uncontrolled de-novo TE insertions. Additionally, the observed increase in γH2Av could also be a consequence of transcriptional upregulation of TEs through formation of R-loops^36^ or to a conflict between retrotransposition and DNA replication^37^. In HeLa cells Line 1 retrotransposon (L1) overexpression causes replication stress by forming stalled replication forks and accumulating cells in S phase^38^; prolonged stalling of these cells in S-phase leads to cell death^38^. In another study, Ardeljain et al also showed that overexpression of L1 triggers DNA damage and stalled replication forks in *p53* deficient cells, and a G1 cell cycle arrest in human epithelial cells expressing p53^37^. These pieces of evidence favour a direct link between TE overexpression, cell cycle arrest and cell death.

Previous experiments performed in Drosophila ovaries and in mammalian cells suggest that dLsd1 regulates TEs by removing active H3K4 methyl marks. Removal of active marks will then promote the methylation of K9 by histone H3K9 methyltransferases, including Egg in Drosophila, and the silencing of TEs^15,39^. ChIP-Seq and/or equivalent experiments in wing discs depleted or not of dLsd1 will help determine the full spectrum of TEs bound by dLsd1 and whether histone marks are affected upon dLsd1 depletion. dLsd1 catalytic dead mutants could also help to establish whether the catalytic activity of dLsd1 is required for TE silencing.

Although our results strongly suggest that the aberrant TE expression observed upon dLsd1 loss affects wing homeostasis, we cannot exclude that genome-wide chromatin accessibility changes due to dLsd1 depletion may also contribute to the wing phenotype. Additionally, our RNA-Seq data analysis showed that dLsd1 regulates directly or indirectly ~ 600 genes, which could also play a role in the phenotypes observed upon dLsd1 depletion in the wings, including size reduction. For example, this analysis highlighted the deregulation of genes that in previous genetic screens were shown to affect wing size, including Hira, Ada, Ac76E^40^, giant and stonewall^41^. In most cases their specific role in controlling tissue size is unknown, underlying the importance of determining how these factors contribute to size determination and whether they are direct targets of dLsd1.

In summary, we discovered that unrestricted TE expression/mobilisation due to loss of dLSD1 affects organ size, laying the foundation to the study of this mechanism in different tissues and/or animals. Interestingly, the mousephenotype.org database reported that *mLSD1*^*+/−*^ mice are born with smaller non-gonadal organs including heart, kidney, liver, lung and spleen suggesting a conserved role of mLSD1 in controlling non-gonadal organ size. Additionally, mutations of the LSD1 partner PHF21A have been described in patients with microcephaly and its knockdown in zebrafish leads to reduced brain size^2^.

Knowing how normal organ size is achieved is crucial for understanding of normal development and has implications for regeneration after injury and for many human pathologies including microcephaly and cancer.

## Methods

### Drosophila melanogaster stocks

Flies were grown on standard Drosophila medium and maintained at 25 °C unless specified otherwise. Experiments were performed using feeding and wandering third-instar larvae. *dLsd1*^*∆N*^ flies were generated by recombination of two piggyBac elements inserted in the *dLsd1* gene^13^. Specifically, the two stocks *w*^*1118*^*; PBac WH Su(var)3-3f00678/TM6B, Tb1* and *hs-Flp/w*^*1118*^*; PBac WH Su(var)3-3f03544/Sb* were mated to generate a precise deletion in the *dLsd1* gene^13^. The resulting mutant has since been maintained in combination with a balancer. *w*^*1118*^ flies used as a wild-type reference in all experiments are maintained as a non-isogenised inbred stock. To remove potential background mutations accumulated on the *dLsd1*^*ΔN*^ chromosome maintained in an inbred balanced stock, new isogenised lines were established: *w*^*1118*^; *dLsd1*^*ΔN*^/TM3, Sb males were mated with *w*^*1118*^ females. *dLsd1*^*ΔN*^/+ females, identified by their yellow eyes due to the mutant allele, were crossed with *w*^*1118*^ males. This cross was repeated over three additional generations. *dLsd1*^*ΔN*^/+ females were then crossed with a TM6B-bearing male, and individual *dLsd1*^*ΔN*^/TM6B, Hu Tb males were used to establish stable lines. Wing sizes of mutant females were comparable to the initial stock, as presented in Supplementary Fig. 3. RNAi efficacy was enhanced by co-expressing Dicer from a UAS-dicer transgene. A complete list of the fly stocks used in this study is available in Supplementary Data 4.

### Organ size measurement and trichome density

Flies used for organ size measurements were reared in uncrowded vials to avoid larval competition and its effects on size. Wings and legs were mounted in Hoyer’s medium. Images were acquired with a wide-field microscope (DM6000) using a 2.5X dry objective. Wing and leg size was quantified using ImageJ. For the head size measurement, adults were anaesthetised with CO_2_ and the head was separated from the rest of the body with a razor blade. Heads were then transferred to slides covered with double-sided sticky tape. A Nikon SMZ18 microscope was used to obtain images and the distance between two macrochaetes positioned between the eyes was measured with *image J*. To determine cell density, trichomes/bristles were counted on images acquired with a wide-field microscope DM6000 using a 40X objective. The number of trichomes in a square of 405,538 pixels or 0.010543 mm^2^ below the posterior cross vein was counted using *ImageJ*. To calculate cell size, the area of the square was divided by the number of trichomes. Cell number and cell size were expressed as the percentage relative to *wild-type* control flies.

### Clonal analysis

Clones were induced by FLP/FRT-mediated mitotic recombination. At 24–48 h after egg laying (AEL) larvae were heat-shocked at 37 °C for 40 min, then harvested ~96 h later at the L3 wandering stage. Wing discs were dissected, fixed and mounted in Vectashield medium containing DAPI (Eurobio-vector). Clone size was measured following the clone contours, identified by the chromosomal GFP marker, with *ImageJ*.

### Immunostaining

Wing discs from feeding and wandering L3 larvae were dissected in cold PBS, fixed in 4% paraformaldehyde (PFA), washed, permeabilised and blocked in blocking solution (PBS/0.2% Triton/1%BSA) for 1 h. Samples were incubated with primary antibodies in blocking solution for 2 h, washed and incubated with Cy3 labelled secondary antibodies (Jackson Immunoresearch) for 1 h. After washing in PBS/0.2% Triton X-100, discs were mounted in Vectashield medium containing DAPI (Eurobio-vector). For L3 wandering L3 wing discs, imaging spacers (0.12 mm depth) were used.

### Immunoblotting

Wing discs were dissected from third instar larvae, washed in PBS and resuspended in E1A Buffer (50 mM HEPES, 250 mM NaCl, 0.1% NP40, 1 mM DTT, 0.2 mM PMSF, Leupeptin, Aprotinin). After homogenisation with a pestle, samples were sonicated and centrifuged. Protein extracts were recovered and quantified using the DC^TM^ protein assay kit (BIORAD). Immunoblots were performed using standard procedure. Blots were developed by photo-luminescence using Super Signal West Dura 34075 (Thermo Scientific) and Chemidoc Touch Imaging System (BioRad).

### EdU labelling

For EdU labelling, the Click it Plus EdU A647 kit (Ref C10640—Lot 1784148—Invitrogen) was used. Wing discs from L3 feeding larvae were dissected and incubated with 30 µM EdU in 200 µL S2 Schneider medium supplemented with 10% foetal bovine serum and 1% penicillin/streptomycin for 30 min at 25 °C. Samples were washed in PBS and fixed with 4% PFA. They were then washed in PBS/ 3%, BSA permeabilized in PBS/0.5% Triton-X-100 for 1 h, and incubated in EdU detection cocktail for 40 min, as recommended by the manufacturer (Invitrogen). Samples were then washed in PBS and mounted in Vectashield medium containing DAPI (Eurobio-vector).

### Imaginal disc image acquisition, treatment and quantification

The confocal Zeiss LSM 710 microscope was used to acquire imaginal disc images with a 40X oil objective lens at 0.8 zoom. For EdU staining, an additional step of deconvolution was performed using Huygens professional. The Apoptotic index and DNA damage index—area of DCP-1-positive cells in µm^2^ and area of ɣH2Av-positive cells per 100 µm^2^, respectively,—were calculated using ImageJ by measuring the area of DCP1 or ɣH2Av positive regions of the projected z-stacks in a defined area of the wing pouch. The mitotic index— number of PH3-positive cells per 100 µm^2^—was calculated using the ICY spots detector plugin and by counting the number of PH3-positive cells in 3D in a defined area of the wing pouch. The S-phase index— number of EdU-positive cells per 100 µm^2^—was calculated by averaging the number of EdU-positive cells on four planes displaying the maximum number of cells in the z-stack using the Huygens professional Advanced Objects Analyser plugin in a defined area of the wing pouch.

### Antibodies

The list of primary antibodies used in this study is provided in Supplementary Data 5. The following secondary antibodies were used: Cy3 anti-mouse, Cy3 anti-rabbit (Jackson Immunoresearch at a concentration of 1:250), anti-mouse HRP and anti-rabbit HRP (Sigma at a concentration of 1:20000).

### RNA extraction and RNA sequencing

Wing discs from L3 wandering female larvae were dissected in ice-cold PBS. RNA was extracted from 30 wing discs per sample using TriZol (Invitrogen) with the addition of RNAsecure (ref AM7006—Ambion). DNA contaminants were eliminated using the DNA-free DNA Removal kit (ref AM1906—Invitrogen). RNA cleanup and purification were performed using the RNeasy Mini Kit (ref 74104—QIAGEN). RNA concentration was assessed with the QUBIT technology (ref Q10210— Thermofisher) and RNA integrity was verified with a BioAnalyzer (ref 5057-1511—Agilent).

Three RNA samples of 500 ng/each per genotype were sent to Fasteris (Switzerland) for poly-A purification and for library preparation using the Illumina TruSeq stranded mRNA Library preparation following the manufacturer’s instruction. Libraries were sequenced using a HiSeq 4000 machine (paired-end 150 bp reads) following the manufacturer’s instructions. All six libraries were multiplexed on the same lane. Reads were then trimmed to obtain paired-end 75 bp reads.

### RT-qPCR

cDNA was obtained from 500 ng of total RNA using TaqMan reverse transcription reagents (ref N8080234 - PE Applied Biosystems). Quantitative PCRs (qPCR) were performed using SYBR Green (FastStart Universal SYBR Green Master—Roche) with CFX96 or CFX384 thermocyclers (Biorad). Quantification was performed using the 2^−∆∆Ct^ method. *RP49*, *Tubulin* and *GAPDH* were used as control for normalisation. All RT-qPCRs were performed at least with biological triplicates. The primer sequences are provided in Supplementary Data 6.

### Chromatin Immunoprecipitation (ChIP)

Six hundred wing discs from the *w*^*1118*^ (wild-type) strain were dissected from L3 larvae and fixed in 1% Formaldehyde for 10 min. The reaction was stopped by glycine addition, and wing discs were resuspended in A1 buffer (60 mM KCl, 15 mM NaCl, 4 mM MgCl_2_, 15 mM HEPES (pH7.6), 0.5% Triton X-100, 0.5 mM DTT in the presence of the cOmplete protease inhibitor cocktail from SIGMA). Samples were homogenised with a Dounce homogeniser, centrifuged and harvested in IP buffer (1%Triton X-100, 0.1% Deoxycholate, 50 mM Tris pH 8.1, 150 mM NaCl, 5 mM EDTA). ChIP experiments were performed as in ref. ^15^ The extracted DNA was used for qPCR. Primer sequences for ChIP-qPCR are provided in Supplementary Data 7.

### DNA Isolation, Oxford Nanopore MinION Sequencing and Base Calling

Genomic DNA was extracted from *wild-type* and *dLsd1* mutant wing discs using the Nanobind tissue kit from Pacific Biosciences (cat#102-302-100). The protocol for low tissue input was followed and adapted for insects: 200 wing discs from both genotypes were homogenised in 50 µl IL buffer with pellet pestle 20–30x, followed by addition of 20 µl Proteinase K and agitated at 900 rpm at 55 °C on a thermomixer for 1 h. Then, 20 µl RNAse A was added for 15 min. Samples were centrifuged at 16,000 × *g* at 4 °C for 5 min. Supernatants were filtered through a 70 µm filter placed in a 1.5 ml Lobind microcentrifuge tube and recovered.

25 µl buffer BL3 was added to samples and mixed 5 times by inversion. Then the Nanobind disc was added followed by the addition of 150 µl isopropanol. Tubes were mixed at room temperature at 20 rpm for 30 min. Samples were washed with 500 µl CW1 and with 500 µl CW2. Elutions were performed in 20 µl buffer EB.

2.5 µl of genomic DNA per sample was amplified with the 4BBTM TruePrime® WGA Kit (4basebio, SKU#350025) and products were purified as recommended by ONT after a whole genome amplification step: 1.5 µg amplified genomic DNA was incubated with T7 endonuclease I (NEB, M0302) and purified with AMPure XP beads prepared in custom buffer (10 mM Tris-HCL pH 8; 0.5 M EDTA pH 8; 1.6 M NaCl; 11% PEG 8000). Elution was performed in 49 µl H2O. The amplified genomic DNA was quantified using a Qubit® 4 Fluorometer (Thermofisher scientific). The totality of genomic DNA (~800 ng) was repaired and end-prepped using the NEBNext Companion Module for ONT Ligation Seq (NEB, E7180). Ligation was then performed using the Ligation sequencing gDNA V14 kit (ONT, SQK-LSK114).

Genomic DNA was extracted from 40 mg of *dLsd1*^*ΔN*^ heterozygous male adults using the Nanobind tissue kit from Pacific Biosciences (cat#102-302-100) and the protocol for DNA extraction with pellet pestle for tissue disruption was followed. The genomic DNA quality and quantity were evaluated using a NanoDrop™ One UV-Vis spectrophotometer (Thermofisher Scientific) and a Qubit® 4.0 Fluorometer (Thermofisher Scientific), respectively. Then 1.8 g of genomic DNA was repaired and end-prepped using the NEBNext Companion Module for ONT Ligation Seq (NEB, E7180).

MinION sequencing was performed on MinION R10.4.1 flow cells (FLO-MIN114, ONT) and a nanopore MinION Mk1B sequencer controlled by the ONT MinKNOW software (v23.04.6). Base calling was performed after sequencing using the GPU-enabled guppy basecaller in high accuracy mode (v6.5.7).

### Genome assembly

The genome of *dLsd1*^*∆N*/+^ adults was assembled according to Mohamed et al.^42^ except that more recent versions were used for Flye (v2.9.3)^43^ and RACON (v1.4.3)^44^ and that, instead of RAGOO, RagTag (v2.1.0)^45^ was used with default parameters and scaffold command. The statistics are listed in Supplementary Data 8.

### Bioinformatic analysis (RNA-Seq)

The RNA-Seq raw data consisted of six (3 *wild-type* and 3 *dLsd1 mutan*t) Illumina stranded paired-end libraries (2*150 bases reads). After the sequencing quality check (fastqc v0.10.1), reads were trimmed based on their quality (trimmomatic v.0.36 with parameters SLIDINGWINDOW:5:28 MINLEN:50). Reads were aligned using STAR (v 2.5.2a) and reads were mapped to the FlyBase reference dm6 genome assembly. Duplicates were removed using Picard (v 2.8.3). Differential expression between wild-type and mutant samples was determined using htseq-count (v 0.6.0) and the R library DESeq2. Transposons differential analysis was done with TEToolkit (v 1.5.1) and the volcano plot was created using the R package ggplot2. Gene ontology analysis was performed with the DAVID (Database for the Annotation, Visualisation and Integrated Discovery) software package (v6.8) (https://david.ncifcrf.gov). Adjusted *p* values were calculated using the Benjamini-Hochberg method.

### Insertional TE detection in *wild-type* and *dLsd1 mutan*t wing discs

The obtained *dLsd1*^*∆N*/+^ genome assembly was the reference genome to map long reads from either the *wild-type* or the mutant wing discs library using TrEMOLO (v2.5.1)^28^.

### Statistics and reproducibility

Data in histograms are presented as the mean ± standard deviation of at least three independent biological replicates or as the median in box plots. Details on the statistical analyses for all experiments can be found in the figure legends. Statistical analyses were done with R or Prism 9 (https://www.graphpad.com/scientific-software/prism/).

## Supplementary information

Supplementary Information

Supplementary Data 1–8

Supplementary Data 9

Description of Additional Supplementary Files

## Data Availability

All the numerical data supporting the finding of this study can be found in the Supplementary Data 9. The raw western blot image is provided in Supplementary Fig. 10. The RNA-seq data were deposited in NCBI GEO (GSE244445). The LR sequencing data used for this study were deposited at ENA under the accession numbers PRJEB67336 and ERP152373. Any remaining information can be obtained from the corresponding author upon reasonable request.
