# Supplementary material for: The histone demethylase dLsd1 regulates organ size by silencing transposable elements
Source: Commun Biol. 2025 Feb 20;8:272. doi: 10.1038/s42003-025-07724-6 (PMC11842725; doi:10.1038/s42003-025-07724-6)
Supplement: Supplementary file 1 — Supplementary Information [file 42003_2025_7724_MOESM1_ESM.pdf]

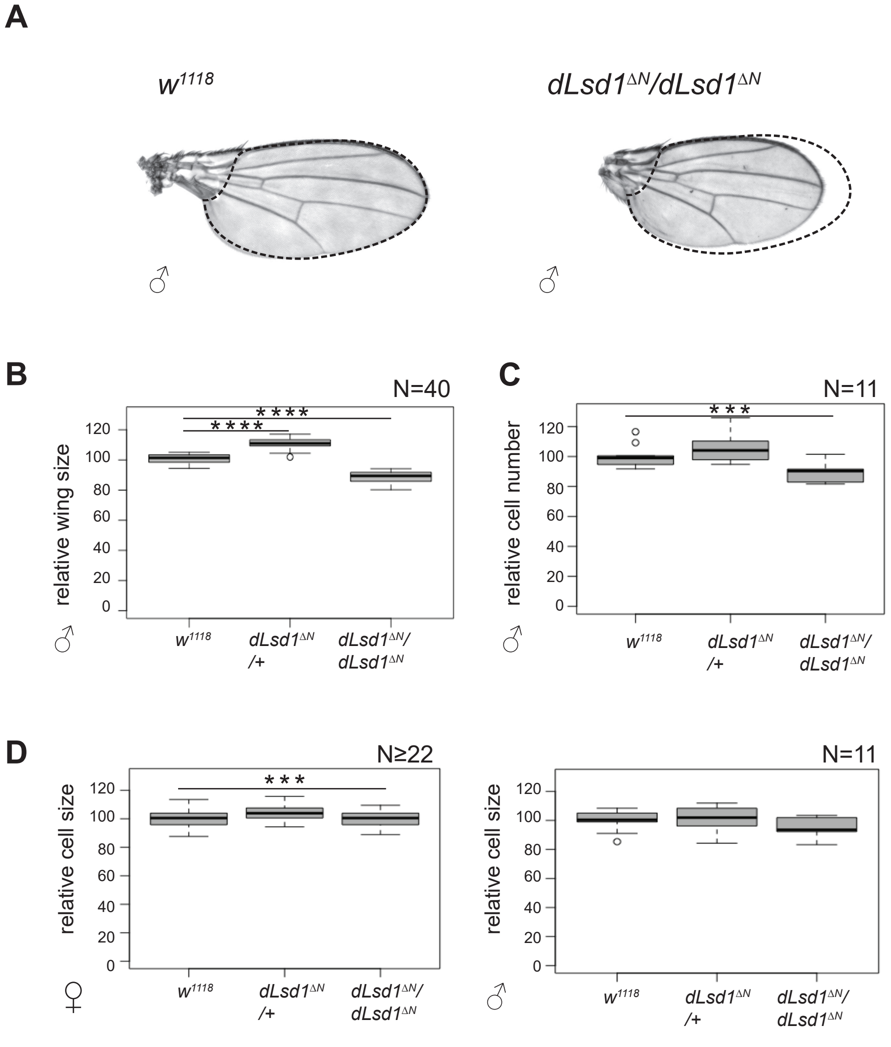

Supplementary Figure 1. dLsd1 loss of function mutation results in the reduction of wing size in males. A) Images of wild-type (*w^{1118}*), *$dLsd1^{\Delta N}$* heterozygous mutant ($dLsd1^{\Delta N}$ /+) and *$dLsd1^{\Delta N}$* homozygous mutant (*$dLsd1^{\Delta N}$/ $dLsd1^{\Delta N}$*) wings of adult males. The black dotted line marks the size of the wild-type wing. **B)** Quantification of wing areas (relative to wild-type) in males of the indicated genotypes. **C)** Quantification of cell number (relative to wild-type) in wings of males of the indicated genotypes. **D)** Quantification of cell size (relative to wild-type) in wings of males and females of the indicated genotypes. N indicates the number of wings counted. *P value < 0.05, **P value < 0.02 , ***P value< 0.01, ****P value < 0.001 (unpaired t-test with Welch correction).

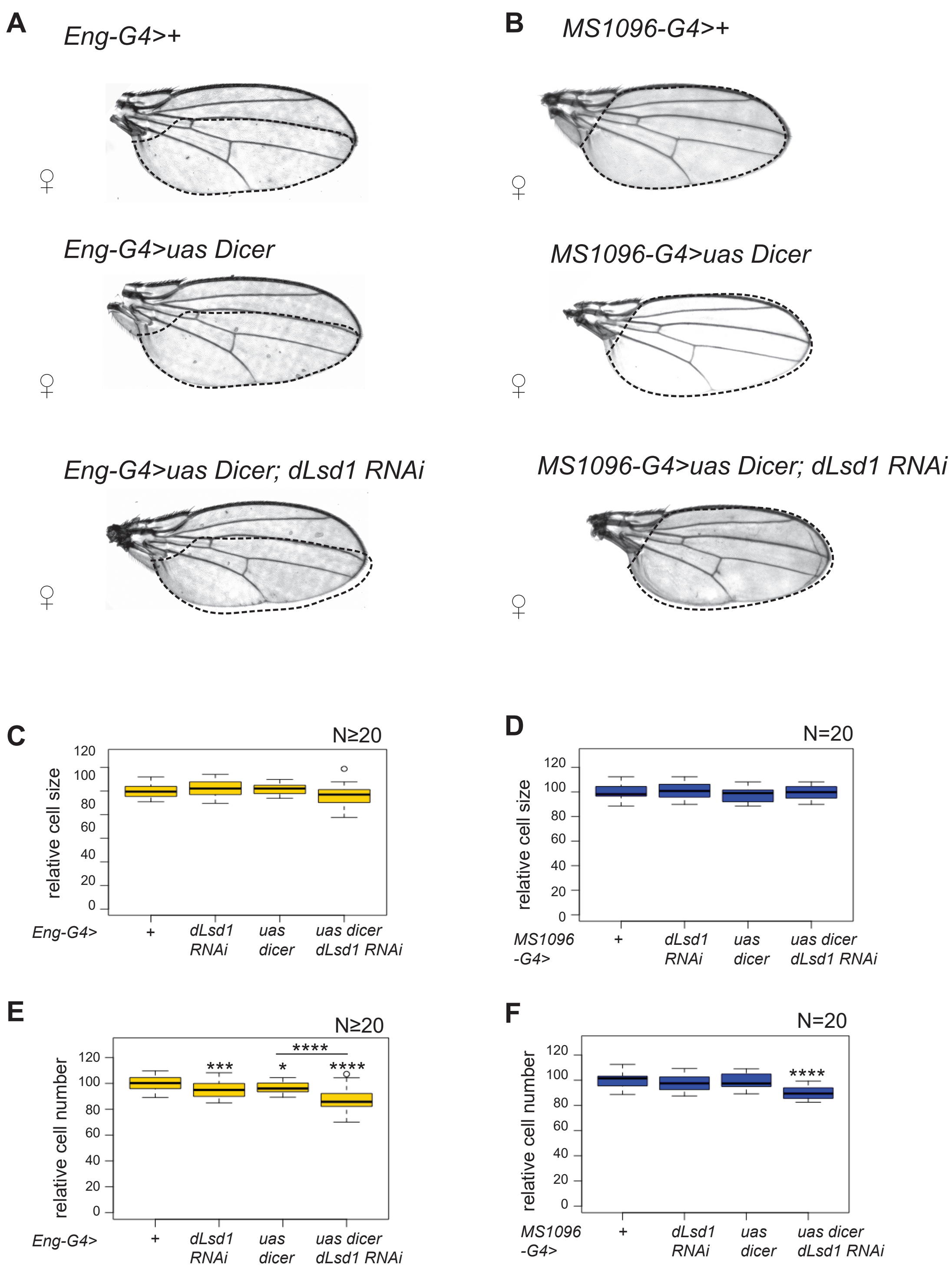

Supplementary Figure 2. dLsd1 depletion reduces the adult wing size. A, B) RNA interference against dLsd1 in the posterior part of the wing decreases wing size in adults compared with control. Representative images of adult wings expressing an RNAi against dLsd1 and/or UAS-dicer under the control of the *Eng-GAL4* driver (A) or the *MS1096-GAL4* driver B). **C, D)** Quantification of cell size (relative to control) in wings from females of the indicated genotypes. **E, F)** Quantification of total cell number (relative to control) in wings from females of the indicated genotypes. Cell number and size were measured in the posterior compartment. N indicates the number of wings counted. *P value < 0.05, **P value < 0.02, ***P value< 0.01, ****P value < 0.001 (unpaired t-test with Welch correction). The circle in the boxplots represents the outliers.

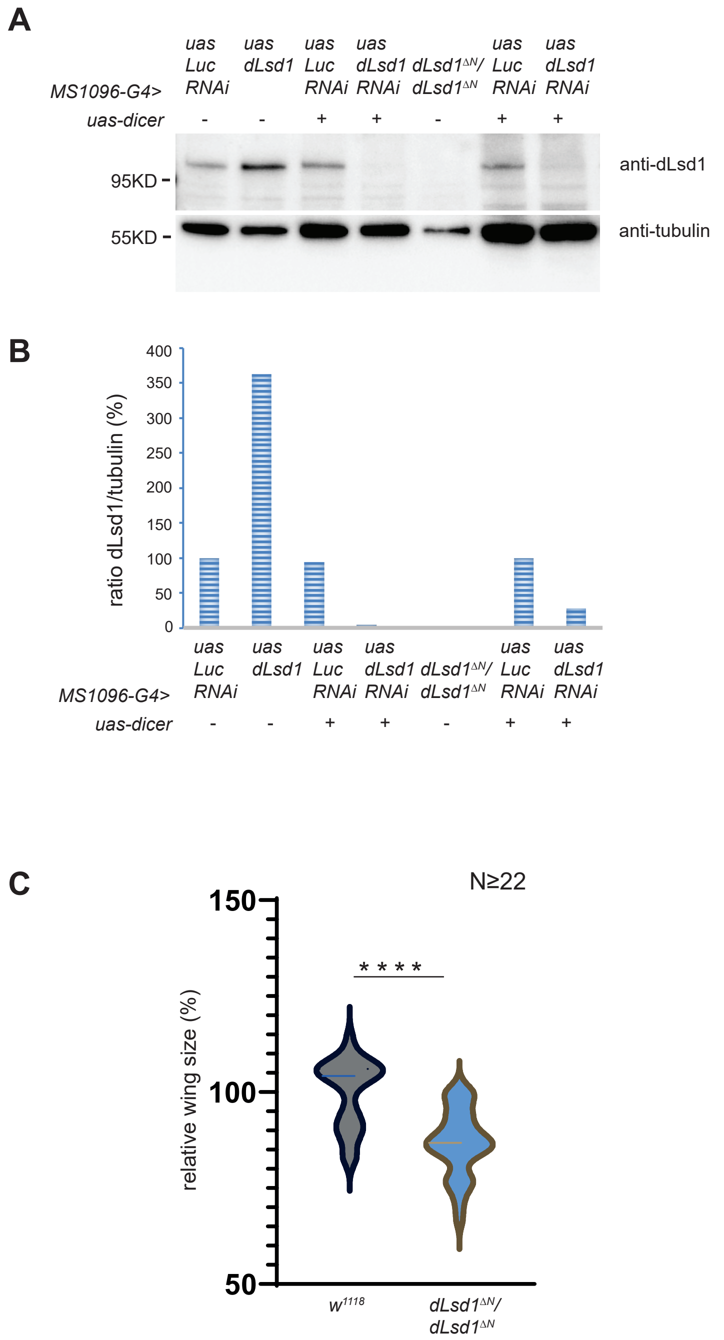

Supplementary Figure 3. Quantification of dLsd1 levels in wing discs. A) Immunoblot showing dLsd1 expression levels in wing discs of the indicated genotypes. Lanes 3 and 4 and lane 6 and 7 are biological replicates. Tubulin was used as loading control. **B)** Quantification of the dLsd1/tubulin ratio. **C)** Quantification of wing size (relative to wild-type) in isogenic females of the indicated genotypes. N indicates the number of wings counted. ****P value < 0.001 (unpaired t-test with Welch correction)

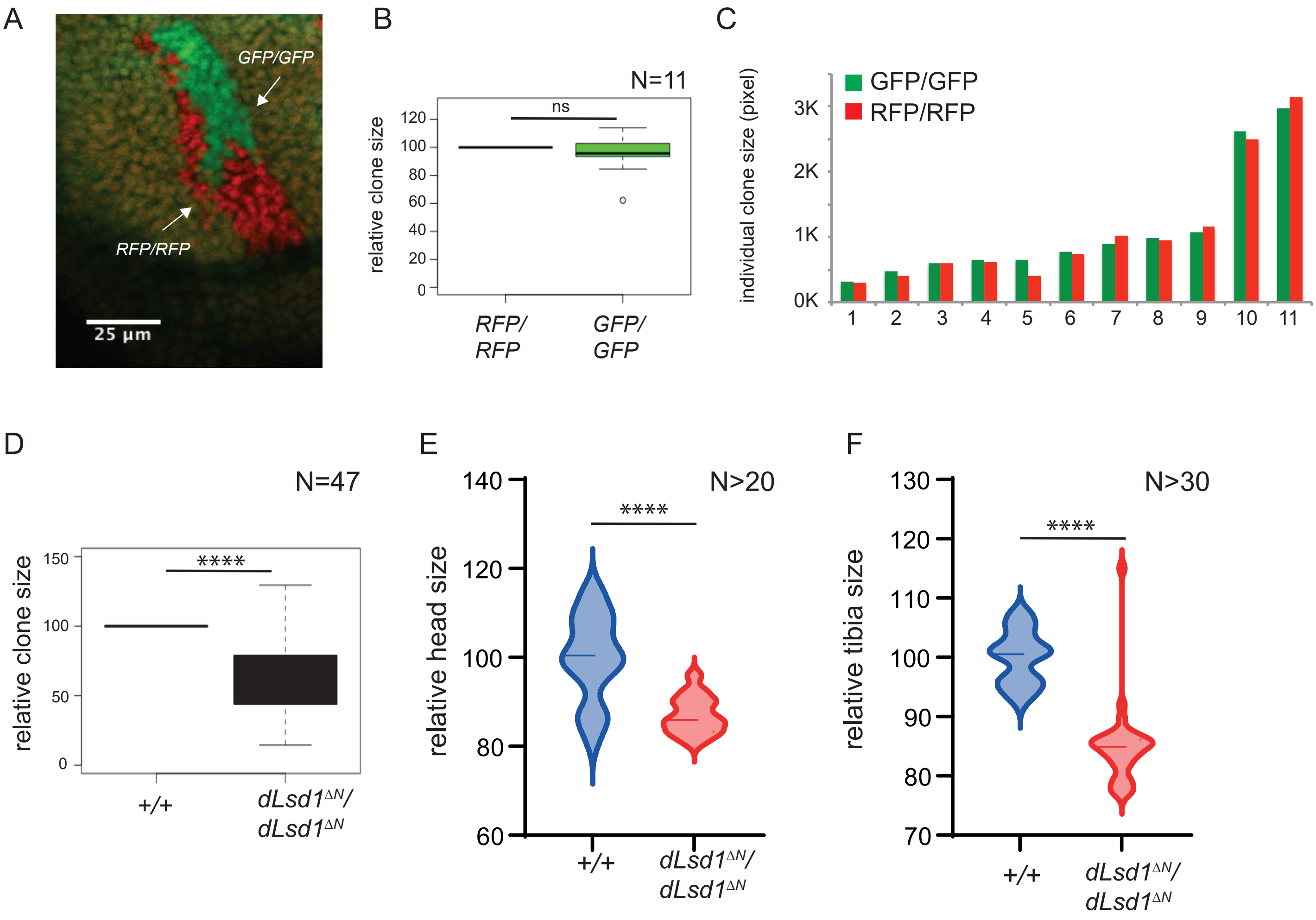

Supplementary Figure 4. The GFP allele does not affect clone size. A) Image of a clone of cells marked by two GFP copies and the twin clone of cells marked by two RFP copies from a wing disc of a third instar larvae. Scale bar: 25mM. **B)** Quantification of mRFP homozygous clone size relative to their GFP homozygous counterparts. N is the total number of twin clones counted. An unpaired t-test with Welch correction did not find any significant difference in size between twin clones. The circle in the boxplot represents the outliers. **C)** Quantification of the size of mRFP homozygous clones and their GFP homozygous twin for each clone in pixels. **D)** Box plot showing the quantification of *dLsd1*$^{\Delta N}$/ *dLsd1*$^{\Delta N}$ clone size relative to their GFP homozygous twin in legs, haltere and eye discs. p-value < 2.2e-16 (Student's t.test). **E)** Violin plot showing the relative head size of adult *dLsd1*$^{\Delta N}$/ *dLsd1*$^{\Delta N}$ and wild type individuals. **F)** Violin plot showing the relative tibia length of adult *dLsd1*$^{\Delta N/}$ *dLsd1*$^{\Delta N}$ and wild type individuals. In E) and F), N indicates the number of individuals and the P value is <0.001 (Mann Whitney U test).

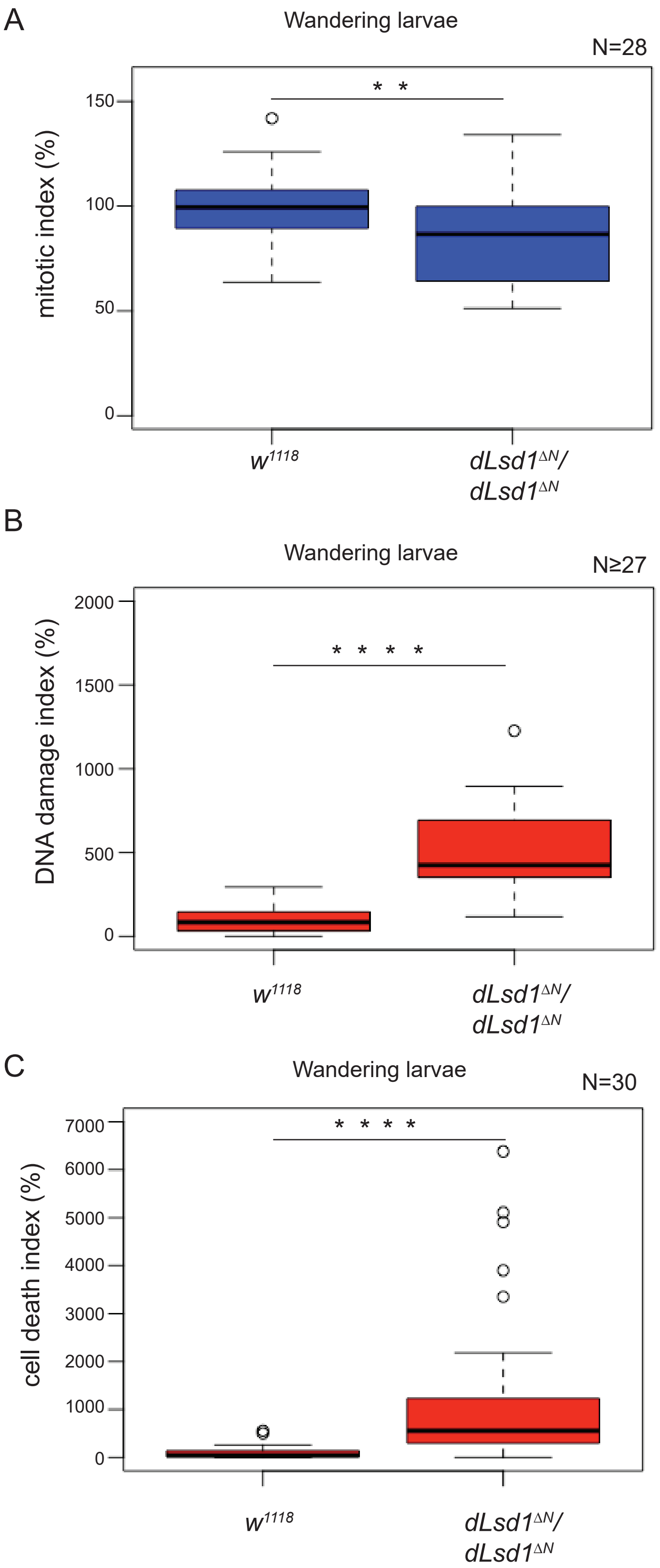

Supplementary Figure 5. The dLsd1 loss of function mutation affects the mitotic index of wandering L3 wing discs and leads to increased DNA DSBs and caspase activation. A) Quantification of the mitotic index in wild-type and *dLsd1*$^{\Delta N}$/ *dLsd1*$^{\Delta N}$ wing pouches of wing discs from wandering L3 females. The mitotic index is expressed as the percentage relative to wild-type control. These experiments were performed in triplicates. N indicates the number of wing discs counted. **B)** Quantification of the DNA damage index in wild-type and *dLsd1*$^{\Delta N}$/ *dLsd1*$^{\Delta N}$ wing pouches of wing discs from wandering L3 females. The DNA damage index is expressed as the percentage relative to wild-type control. These experiments were performed in triplicates. **C)** Quantification of the cell death index in wild-type and *dLsd1*$^{\Delta N}$/ *dLsd1*$^{\Delta N}$ wing pouches of wing discs from wandering L3 females. The cell death index is expressed as the percentage relative towild-type control. These experiments were performed in triplicates. *P value < 0.05, **P value < 0.02, ***P value< 0.01, ****P value < 0.001 (Student's t-test).

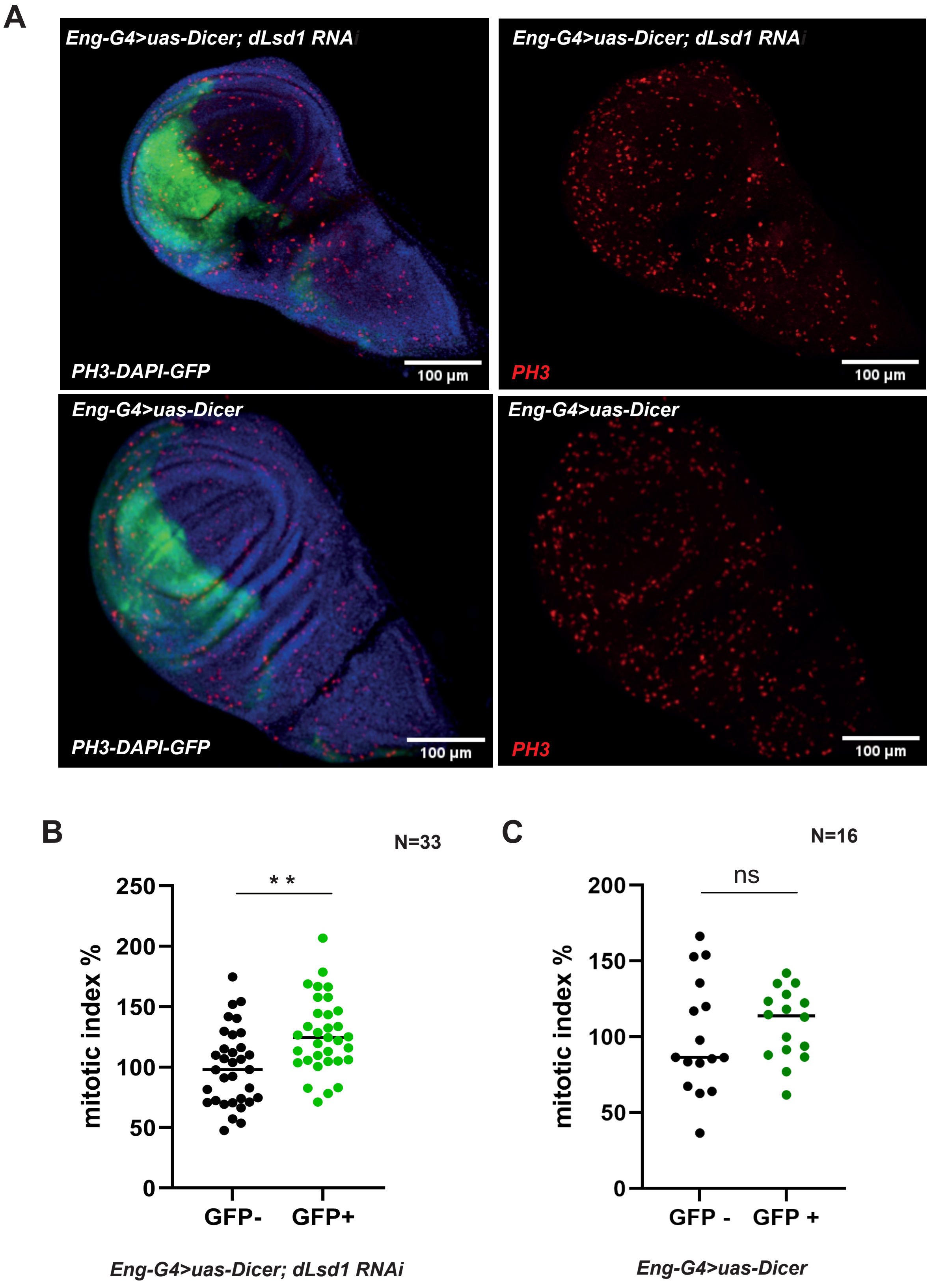

Supplementary Figure 6. dLSD1 RNAi perturbs the cell cycle. A) Representative image of wing discs of the indicated genotypes. Discs were stained with DAPI to visualize DNA and with PH3 to mark cells in mitosis. GFP marks the posterior compartment. **B)** Quantification of the mitotic index in the anterior (GFP-) and posterior (GFP+) compartments of the wing pouch upon *Eng-G4* driven depletion of dLsd1 by RNAi. The P value was determined by performing a Mann Whitney test (P value=0.0013). **C)** Quantification of the mitotic index in the anterior (GFP-) and posterior (GFP+) compartments of the wing pouch of control flies. The P value was determined by performing a Mann Whitney test (ns= non statistically significant, P value=0.3).

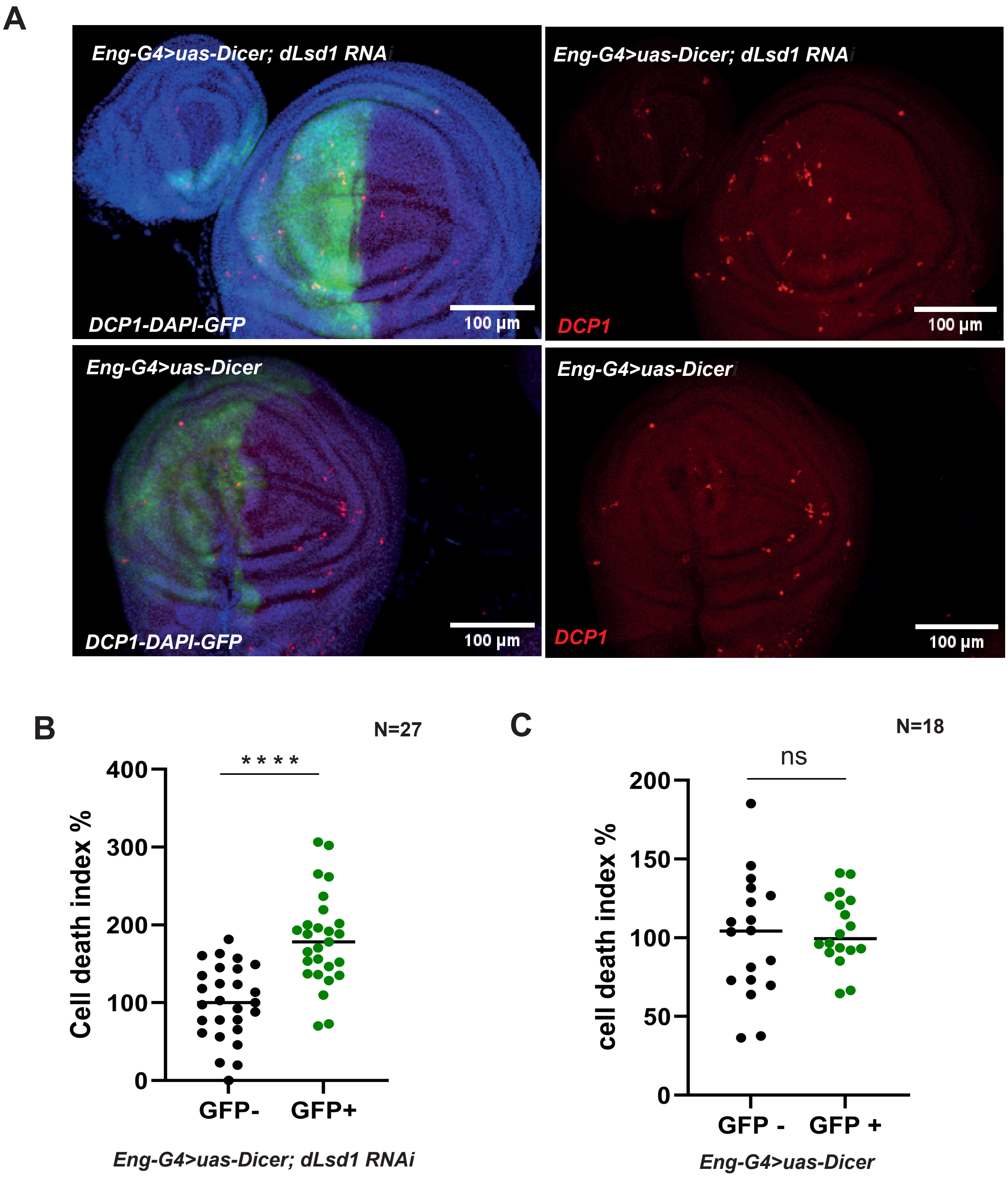

Supplementary Figure 7. dLSD1 RNAi induces an increase in cell death. A) Representative image of wing discs of the indicated genotypes. Discs were stained with DAPI to visualize DNA and with DCP-1 to mark apoptotic cells. GFP marks the posterior compartment. **B)** Quantification of the cell death index in the anterior (GFP-) and posterior (GFP+) compartments of the wing pouch upon *Eng-G4* driven depletion of dLsd1 by RNAi. N indicates the number of wing discs used in each experiment. P values were determined by performing a Mann Whitney test (****P value < 0.001). **C)** Quantification of the cell death index in the anterior (GFP-) and posterior (GFP+) compartments of the wing pouch of control flies. The P value was determined by performing a Mann Whitney test (ns= non statistically significant, P value=0.6)

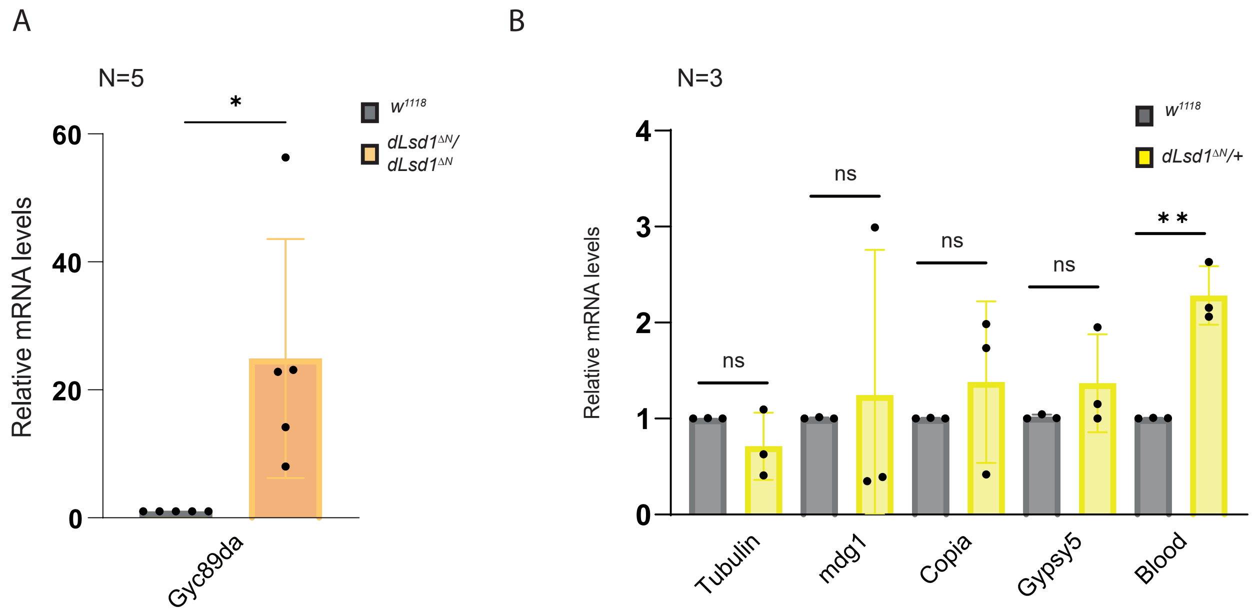

Supplementary Figure 8. The dLsd1 mutation strongly affects the expression of Gyc89Da. A) RT-qPCR analysis of the expression of Gyc89Da in wild-type and *dLsd1*$^{\Delta N}$*/ dLsd1*$^{\Delta N}$ wing discs of L3 wandering females. The expression level was relative to wild-type control and Rp49 was used as a reference. Error bars indicate the standard deviation. N indicates the number of biological replicates. *: P value < 0.05 (Student's t-test). **B)** RT-qPCR analysis of the expression of the indicated genes and TEs in wild-type and *dLsd1*$^{\Delta N}$ /+ wing discs from L3 wandering females. The expression level was relative to wild-type and Rp49 was used as a reference. Error bars indicate the standard deviation. N indicates the number of biological replicates. **: P value= 0.002, ns, not significant (Student's t-test).

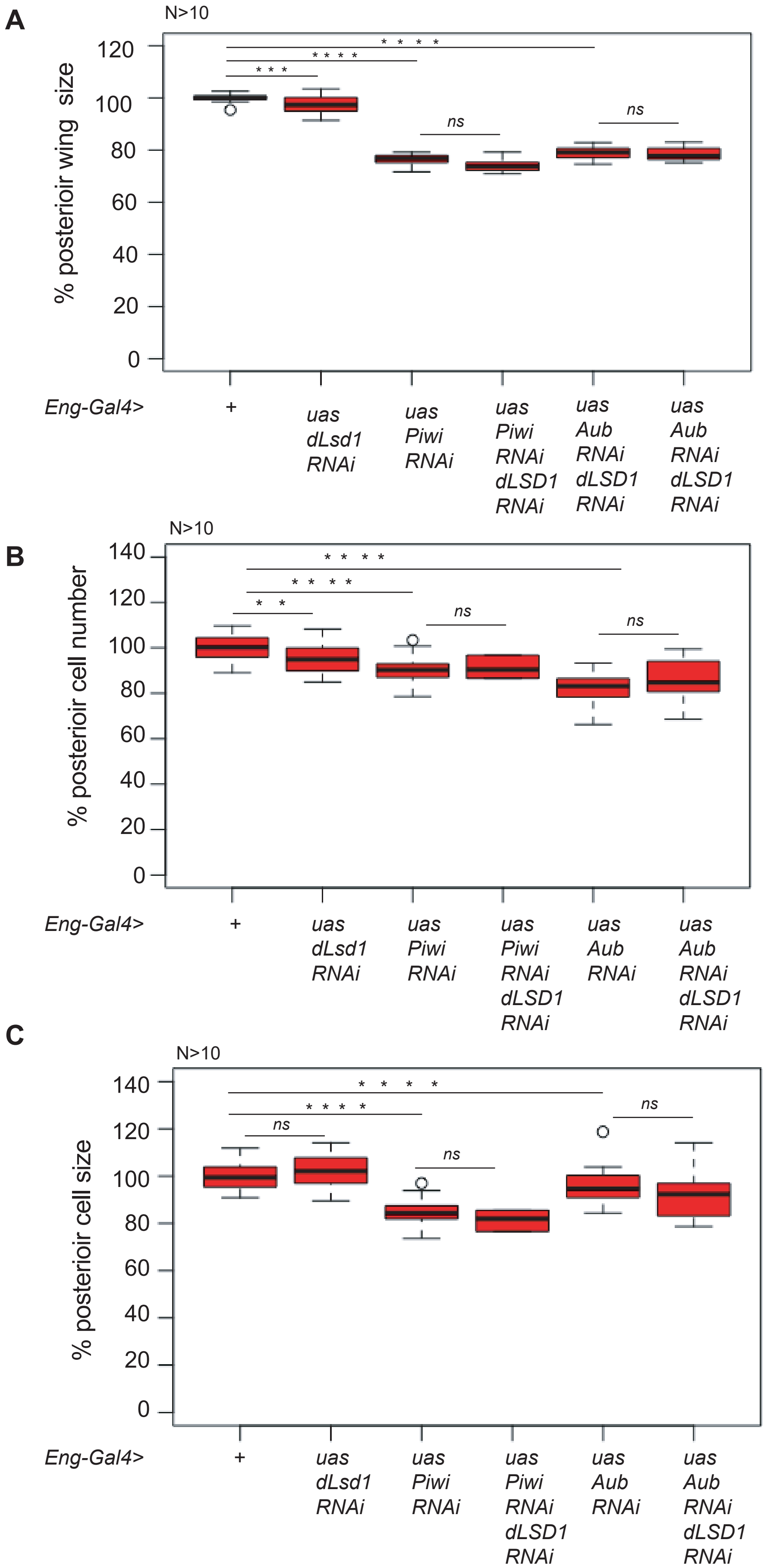

Supplementary Figure 9. Effect of Piwi and Aubergine depletion on wing size and cell number in wing discs. A) RNAi based silencing of Piwi and Aubergine results in smaller wing size.Quantification of the posterior wing areas from females of the indicated genotypes. * P value < 0.05, ** P value < 0.02, *** P value < 0.01, **** P value < 0.001, ns not significant (Mann Whitney U test). **B)** Quantification of total wings cell number in females of the indicated genotypes. **C)** Quantification of wing cell size in females of the indicated genotypes. Total cell numbers and cell sizes are expressed as the percentage relative to wild-type. N indicates the number of wings counted. An was performed to indicate significance *P value < 0.05, **P value < 0.02 , ***P value< 0.01, ****P value < 0.001 (unpaired t-test with Welch correction).

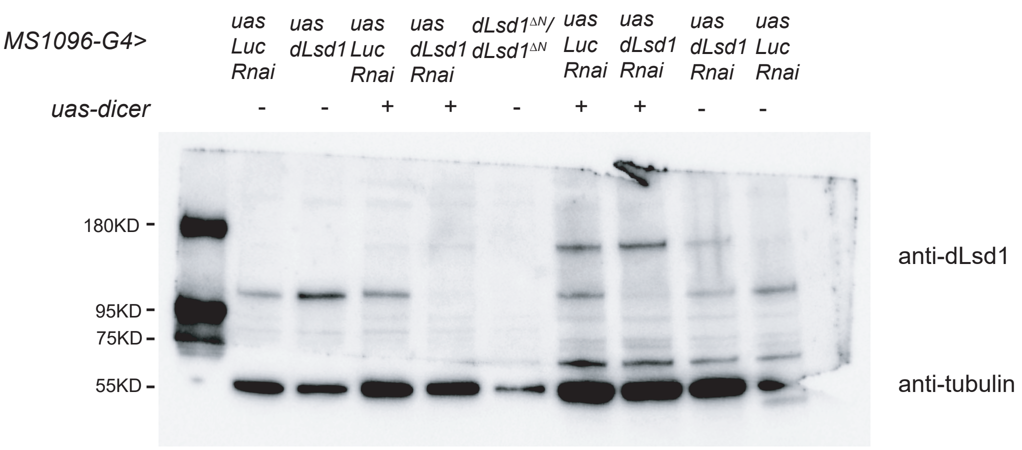

Supplementary Figure 10. Raw image of the immunoblot shown in Supplementary Figure 3.
