# Supplementary material for: The histone demethylase dLsd1 regulates organ size by silencing transposable elements
Source: Commun Biol. 2025 Feb 20;8:272. doi: 10.1038/s42003-025-07724-6 (PMC11842725; doi:10.1038/s42003-025-07724-6)
Supplement: Supplementary file 4 — Description of Additional Supplementary Files [file 42003_2025_7724_MOESM4_ESM.pdf]

Description of Additional Supplementary Files

File name : Supplementary Data 1

Description : List of upregulated and downregulated genes

File name : Supplementary Data 2

Description : List of upregulated and downregulated Transposable Elements

File name : Supplementary Data 3

Description : List of new TE insertions

File name : Supplementary Data 4

Description : Fly lines used in this study.

File name : Supplementary Data 5

Description : Primary antibodies used in this study

File name : Supplementary Data 6

Description : Sequences of the RT-qPCR primer used in this study

File name : Supplementary Data 7

Description : Sequences of the ChIP-qPCR primers used in this study

File name : Supplementary Data 8

Description : Statistics of the libraries. All lengths are expressed in bases

File name : Supplementary Data 9

Description : Numerical source data
